# Review and Perspectives on the Structure–Function Relationships of the Gag Subunits of Feline Immunodeficiency Virus

**DOI:** 10.3390/pathogens10111502

**Published:** 2021-11-18

**Authors:** Mathieu Long, Johan Toesca, Christophe Guillon

**Affiliations:** 1 Retroviruses and Structural Biochemistry, Molecular Microbiology and Structural Biochemistry, CNRS, Univ Lyon, UMR5086, 69007 Lyon, France; mathieu.long@ens-lyon.fr (M.L.); johan.toesca@gmail.com (J.T.); 2 Center for Molecular Protein Science, Department of Chemistry, Lund University, Lund, 221 00 Scania, Sweden; 3 Enveloped Viruses, Vectors and Immunotherapy, CIRI-Centre International de Recherche en Infectiologie, Univ Lyon, Université Claude Bernard Lyon 1, UMR5308, ENS Lyon, 69007 Lyon, France

**Keywords:** Feline Immunodeficiency Virus, FIV, Human Immunodeficiency Virus, HIV, Gag, matrix, capsid, nucleocapsid, late domain, structure

## Abstract

The Gag polyprotein is implied in the budding as well as the establishment of the supramolecular architecture of infectious retroviral particles. It is also involved in the early phases of the replication of retroviruses by protecting and transporting the viral genome towards the nucleus of the infected cell until its integration in the host genome. Therefore, understanding the structure–function relationships of the Gag subunits is crucial as each of them can represent a therapeutic target. Though the field has been explored for some time in the area of Human Immunodeficiency Virus (HIV), it is only in the last decade that structural data on Feline Immunodeficiency Virus (FIV) Gag subunits have emerged. As FIV is an important veterinary issue, both in domestic cats and endangered feline species, such data are of prime importance for the development of anti-FIV molecules targeting Gag. This review will focus on the recent advances and perspectives on the structure–function relationships of each subunit of the FIV Gag polyprotein.

## 1. Introduction

Feline Immunodeficiency Virus (FIV) is a retrovirus belonging to the lentivirus genus and infects both domestic and wild feline species. FIV is one of the few non-primate lentiviruses that induces an acquired immunodeficiency syndrome in its natural host and is, as such, one of the closest biological models in small animals for Human Immunodeficiency Virus (HIV) infection [1]. FIV is also considered an attractive lentiviral vector system because of its ability to transduce cells in non-replicative phases [2].

FIV infects domestic cats, but also wild feline species such as lions, hyenas, cheetahs, or pumas, representing an issue both for domestic veterinary practice and wildlife preservation [3]. It is estimated that 4 to 12% of domestic or wild felines are infected, with a disparity depending on the infected species and the countries considered. Inter-animal transmission of the virus is mostly mediated by bites or coitus [4,5]. Infected animals will develop a progressive (5–10 years) immune dysfunction caused by the depletion of CD4^+^ T lymphocytes [2], resulting in the appearance of opportunistic infections, neurological disorders, or neoplasms in infected animals. A vaccine (Fel-o-Vax) has been developed for domestic cats but is only efficient against some of the FIV subtypes. It is therefore only used in a small number of countries where theses subtypes represent the majority of the circulating strains [6]. Hence, specific therapeutic strategies against FIV are needed to address this veterinary issue.

## 2. The Importance of the Gag Polyprotein for the Replication of Lentiviruses

**Figure 1:**
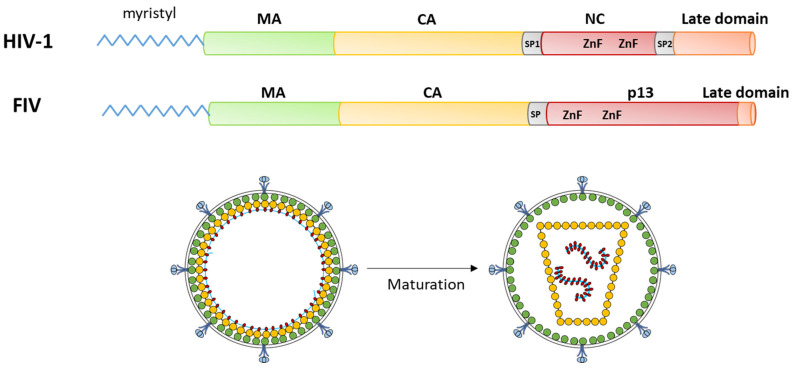
Overview of Gag protein organization and function. Top: organization of the subunits in the frame of the Gag polyprotein: Bottom: organization of the viral particle before (left, immature form) and after (right, mature form) cleavage of the Gag polyprotein in its individual subunits by the viral protease. Subunits are colored all along the scheme as follows: green: MA; yellow: CA; red: NC; pink: late domain; and gray: spacer peptides. Viral RNA in the viral particle is depicted as a thin blue line.

Among the viral targets of potential therapeutic interest, the Gag polyprotein has been scrutinized intensively in the HIV-1 model. Gag is a precursor polyprotein composed of several functional subunits: the matrix protein (MA), which allows the binding of Gag to the plasma membrane through a basic domain and an N-terminal myristoyl moiety anchoring Gag to the membrane; and the capsid protein (CA) that oligomerizes to form the viral core protecting the viral RNA, which is selected and encapsidated through interactions with the nucleocapsid domain (NC) of Gag. Other domains have been identified in HIV-1 Gag, in particular, the spacer peptide SP1 between CA and NC and a C-terminal “late domain”, both seeming to be present in FIV Gag (Figure 1).

The mechanisms of action of Gag have been well defined for HIV-1: the assembly of a new viral particle is initiated by the interaction of NC with the viral RNA [7] with a role of the “late domain” in selecting the correct genomic RNA [8]. This triggers the oligomerization of the Gag polyprotein with the SP1 spacer forming a six-helix bundle [9]. This radial oligomerization is further stabilized by CA/CA [9,10] and MA/MA interactions (Figure 1); the latter resulting in the exposure of the myristoyl group by an “entropic switch” mechanism, increasing the interaction with the inner leaflet of the plasma membrane [11]. This oligomerization results in the assembly of an immature viral particle, which is released through the interaction of the “late domain” with the ESCRT machinery of the cell [12]. Then, the Gag polyprotein is cleaved in its different subunit by the viral protease, resulting in a spatial reorganization of the Gag subunits in the viral particle (Figure 1) [13]. The most flagrant reorganization concerns the viral core, as CA subunits spatially rearrange after their release, transforming the spherical, immature, noninfectious viral core into a fullerene-shaped mature capsid. Notably, this maturation is necessary for the virion to be infectious.

Interestingly, despite only ~20% of sequence identity between HIV and FIV Gag polyprotein, most of these mechanisms have been conserved between FIV and HIV-1. 

The structure–function relationships of the Gag subunits have been extensively studied in the HIV-1 model in order to develop anti-Gag molecules [14,15,16,17]. This has resulted in the recent clinical trial of an anti-HIV molecule targeting Gag assembly [18]. Although the FIV Gag polyprotein is also composed of MA, CA, and NC subunits that are cleaved during maturation [19], the structure–function information concerning FIV Gag are scarce, as the first structural data on Gag subunits have only been emerging in the last decade [20,21,22]. In this review, we will describe briefly the latest data and perspectives concerning the structure–function relationships of FIV Gag subunits.

## 3. FIV MA

**Figure 2:**
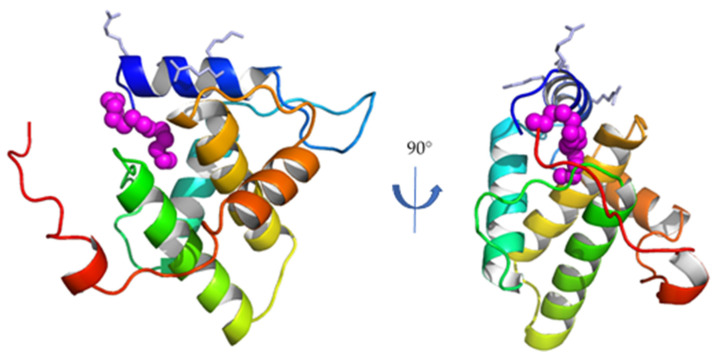
Crystal structure of the monomeric full-length FIV MA (PDB ID 4IC9). Secondary structure elements are colored blue-to-red from the N-to the C-terminus. Side chains of the residues participating to the N-terminal basic patch are displayed in light blue; the myristoyl group is displayed in magenta (adapted from [20]).

The matrix subunit MA is the N-terminal subunit of Gag (Figure 1), and is composed of 131 residues for FIV [23]. It is the first subunit of FIV Gag whose structure was solved (Figure 2) [20]. 

As its HIV-1 homologue, FIV MA is composed of five α-helices. Similarly, it presents a conserved basic patch on its N-terminal helix, oriented towards the putative interaction site with the plasma membrane [20], and a myristoyl group trapped in a hydrophobic cavity in the monomeric form of FIV MA, as demonstrated both by molecular docking and NMR studies [20,24,25]. For HIV-1, it has been suggested that MA/MA interactions mediated by the oligomerization of the Gag polyprotein induce conformational changes that expose this myristoyl group outside the hydrophobic cavity, making it available to interact with the plasma membrane together with the basic patch of helix h1 [11]. Moreover, the reorganization of the MA lattice after the cleavage of HIV-1 Gag also retains the exposure of the myristoyl group and its interaction with the plasma membrane of the infectious particle [26].

Although the exposure of the myristoyl group of FIV MA is necessary for an efficient assembly of FIV, there are currently no structural data or experimental evidence of such an entropic switch [24,25,27]. However, the combination of the basic region of helix h1 with the aliphatic myristoyl chain is likely to mediate the strong anchoring of FIV MA to the inner leaflet of the plasma membrane, as has been described for HIV-1 [28,29], bringing the whole assembly machinery of the Gag polyprotein into the vicinity of the budding site of the particle. 

Membrane targeting of HIV-1 MA involves the direct interaction of MA with specific components of the inner leaflet of the plasma membrane, in particular, phosphatidylinositol-(4,5)-bisphosphate (PI(4,5)P2) [30,31]. Indeed, HIV-1 myristoylated MA targets PI(4,5)P2-rich domains and Gag assembly can even increase PI(4,5)P2 clustering at the membrane [32]. Oppositely, depletion of PI(4,5)P2 from the membrane inhibits the release of HIV-1 virions [31]. Finally, a direct interaction of HIV-1 MA with PI(4,5)P2 after the maturation of the viral particle has been documented [26].

The information available as to the role of PI(4,5)P2 in FIV replication are less exhaustive, but it has been demonstrated that the release of FIV particles is inhibited by depletion of PI(4,5)P2 [24]. This suggests that, as for HIV-1, PI(4,5)P2/ MA interactions are crucial for FIV replication.

Aside from its role in viral assembly, HIV-1 MA has also been implied in the recruitment of envelop glycoproteins at the surface of the virus [33]. This has not been experimentally demonstrated for FIV MA, although it is likely that MA possesses a similar role [34,35], which could represent an interesting therapeutic target against both HIV-1 and FIV.

## 4. FIV CA

**Figure 3:**
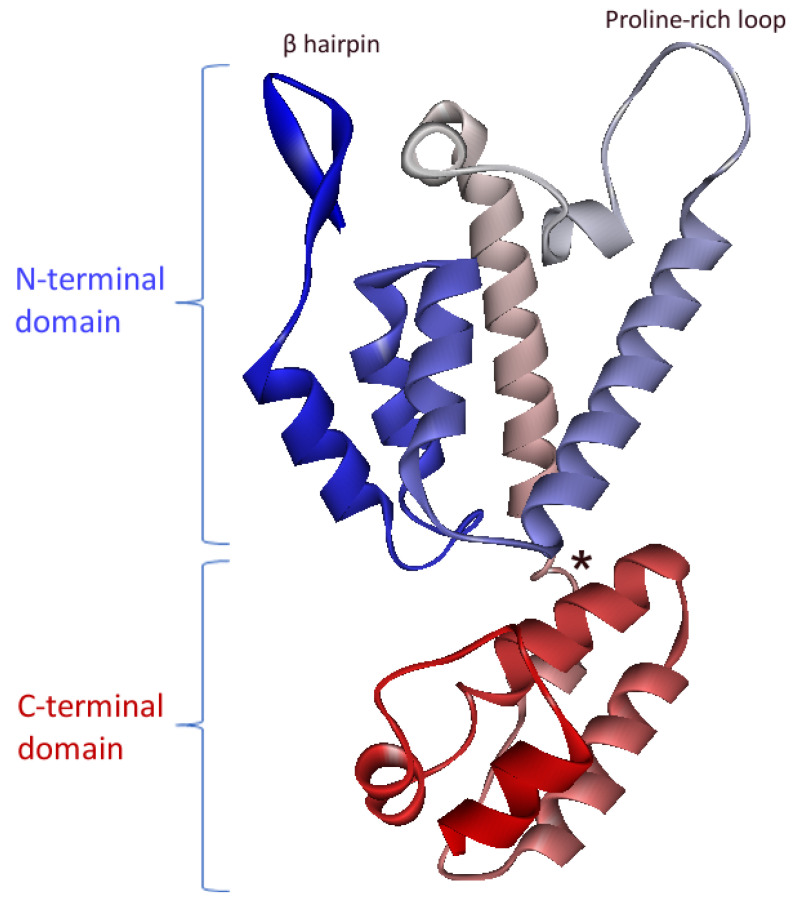
Crystal structure of the monomeric FIV CA (PDB ID 5NA2). Secondary structures are colored blue-to-red from the N- to the C-terminus. The flexible linker between N- and C-terminal domains is indicated by a star (adapted from [22]).

The CA subunit is located directly downstream of MA in the FIV Gag polyprotein (Figure 1). The structure of its C-terminal domain was first solved before the structure of the complete protein was obtained [21,22]. HIV-1 is composed of two domains, N- and C-terminals, separated by a flexible linker. The C-terminal domain contains five α-helices, while the N-terminal domain contains seven α-helices, a large proline-rich loop, and an N-terminal β-hairpin (Figure 3).

**Figure 4:**
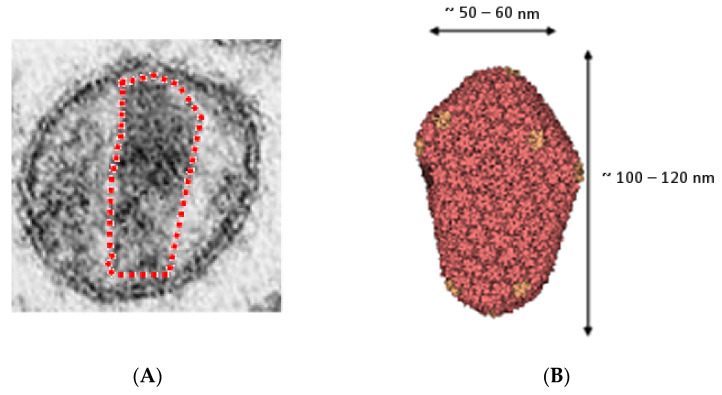
(**A**) Transmission electron micrograph of an HIV-1 mature viral particle with the viral core contoured with a red dotted line; (**B**) Cryo-electron microscopy reconstruction (PDB ID 3J3Q) of the fullerene-shaped viral core with p24 pentamers (orange) and hexamers (red).

In the mature viral particle, between 1500 and 4000 CA subunits will assemble to form a fullerene-shaped conical viral core, which is typical of lentiviruses and can be identified by transmission electron microscopy [36]. For HIV-1, it has been demonstrated that this conical shape derives from the assembly of hexamers and pentamers of CA [36,37,38] (Figure 4). The structures of these pentamers and hexamers of HIV-1 CA have been solved by X-ray crystallography and solid-state NMR [39,40]. 

For FIV, a fullerene-shaped core has also been observed [41]. It has therefore been inferred that the FIV core had similar assembly constraints, and that its formation is likely to involve pentamers and hexamers of FIV CA. However, the structures of these FIV oligomers have yet to be identified [42].

The role of this viral core has been clearly demonstrated in the HIV-1 model and is two-sided: on the one hand, it allows for the protection of the genomic viral RNA from cellular RNAses during the early steps of viral replication, allowing the reverse transcription into double stranded DNA to take place into this core [43,44,45]. On the other hand, after being transported towards the nucleus of the infected cell through the interaction with nucleoporins [46,47,48,49], the core must disassemble to allow for an efficient integration of this reverse-transcribed DNA [50]. Thus, interactions between and/or within CA oligomers in the viral core must be tight enough to protect viral RNA but loose enough to allow spontaneous disassembly prior to integration. The crucial importance of these interactions made the CA an interesting therapeutic target against retroviral infection [16,17,51]. For HIV-1, this has led to the development of inhibitors, including one (GS-6207), which is currently under clinical trial as lenacapavir [18]. Interestingly, GS-6207 binds at the CA/CA interface within HIV-1 CA hexamers and has been optimized from lead molecules using the structural data available on these hexamers [14,52,53]. For FIV, assembly inhibitors have recently been identified that bind the same region of CA, which seems to be similarly able to interact with feline nucleoporins [42,54] and thus represent an interesting therapeutic target. Obtaining structural data on FIV CA oligomers will therefore be of prime importance for the optimization of these compounds towards efficient anti-FIV molecules. 

Of note, the viral core composed of CA proteins is the target of innate antiviral restriction factors. Factors from the tripartite motif protein family (TRIM) will control retroviral infection by binding to the assembled viral cores and inducing their rapid degradation at the early steps of replication [55]. TRIM restriction factors from one species will restrict replication of lentiviruses from other species; for example, human TRIM5α inhibits the replication of murine retroviruses, while rhesus macaque TRIM5α inhibits human immunodeficiency virus type 1 (HIV-1) or FIV infection [56,57]. Oppositely, binding of human cyclophylin A (CypA) to the proline-rich loop of HIV-1 CA blocks the restriction by TRIM5α in human cells, allowing the efficient replication of HIV-1 in its natural host [58]. Of note, FIV CA is also able to bind CypA [59]. However, feline cells do not express restriction factors from the TRIM family [56]. The conservation of CypA binding on FIV CA despite the absence of the endogenous selection pressure by TRIM factors in feline cells is puzzling and deserves further investigations.

## 5. FIV SP

**Figure 5:**
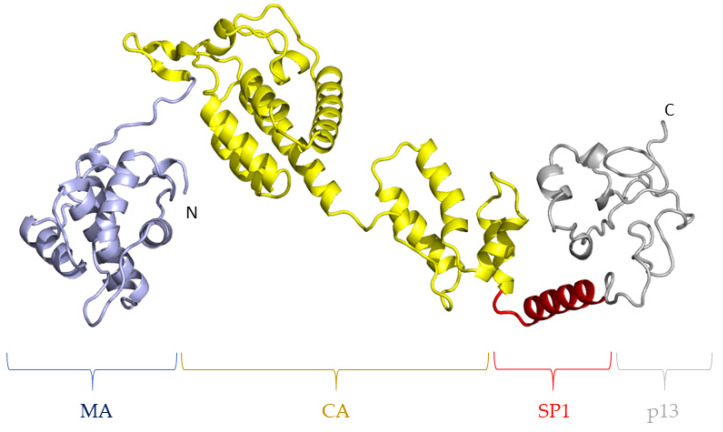
Structure prediction of the FIV Gag polyprotein. From left to right, blue: MA; yellow: CA; red: SP; and gray: p13. N- and C-terminal extremities are indicated. This prediction de novo was performed on the FIV Petaluma sequence (UniProtKB P16087) using the RoseTTAFold server [65].

In HIV-1, the spacer peptide SP1 between the CA and NC subunits has been identified as being necessary to initiate the hexamerization of CA [60]. Indeed, electron cryotomography studies have demonstrated that SP folds as an α-helix and oligomerization of the Gag polyprotein leads to the formation of a 6-helix bundle of the SP regions in the hexamer [9]. Interestingly, SP1 is the target of an anti-HIV molecule named bevirimat, which seems to act by the rigidification of the CA-SP1 boundary, which renders it insensitive to proteolytic cleavage by the viral protease in vitro [61,62]. However, the natural polymorphism of SP1 as well as the appearance of escape mutations hamper the antiviral efficacy of bevirimat against HIV-1 [63,64]. To our knowledge, no experimental structure of the equivalent SP peptide in FIV Gag has been described. However, a de novo prediction of the structure of the complete FIV Gag polyprotein using the RoseTTAFold server [65] suggests that FIV SP is prone to fold as an α-helix and might therefore participate as a six-helix bundle (Figure 5). It is therefore likely that, as for HIV-1, FIV SP is necessary for a correct oligomerization of CA to form the viral core. Specific structural information on FIV SP should help confirm this hypothesis. In particular, our structure prediction suggests that the CA-SP boundary in FIV Gag is flexible and could represent an interesting target for a therapeutic strategy aiming at the stabilization of SP to inhibit FIV Gag maturation.

## 6. FIV p13

For HIV-1, it has been shown that the C-terminus of the Gag polyprotein is composed of two subunits, NC and p6 (or “late domain”), cleaved by the viral protease during maturation, which are respectively involved in viral RNA binding and recruitment of the cellular machinery involved in the abscission of the viral particle [7,8,12,66]. For FIV, the C-terminal region of FIV Gag, named p13, is supposed to assume the same functions. A cleavage site has been suggested in FIV p13, which would result in the release of a small p2 subunit as the “late domain” [19]. However, it is not clear whether this fragment represents an actual structural entity per se. This is why we will describe the structure–functions of the NC and “late domain” of FIV in the frame of the p13 subunit.

**Figure 6:**
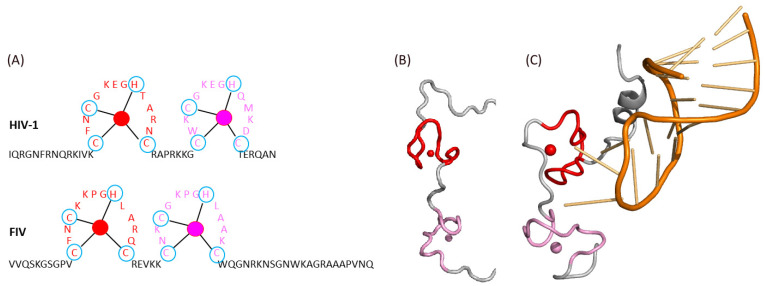
Structure–function of NC: (**A**) comparison of HIV-1 and FIV NC sequences and zinc finger domains (in red and pink with Zn^2+^ ions depicted as plain circles). (**B**,**C**) NMR structure of HIV-1 NC alone (**B**, PDB ID 5I1R) or in complex with the viral RNA (**C**, PDB ID 1A1T). Zinc fingers and Zn^2+^ ions are colored as in (**A**).

Lentiviral NC is necessary for the binding to viral RNA. This ability to bind nucleic acids is due to the presence of two zinc finger domains (ZF) in NC, containing the sequence Cys-X2-Cys-X4-His-X4-Cys, which is conserved between HIV-1 and FIV (Figure 6A) [67]. These zinc fingers form hydrophobic trays that are important for the interaction with viral RNA, while the aromatic residues of each zinc finger allow for the stabilization of these interactions [68]. Notably, the position of the phenylalanine residue is conserved in the first ZF of both HIV-1 and FIV NC, while the position of the tryptophan residue of the second ZF is opposite between these two viruses. Moreover, HIV-1 NC possesses a long N-terminal extremity rich in basic residues, while this is characteristic of the C-terminus of FIV NC (Figure 6A). Notably, this N-terminal extension of HIV-1 NC folds as an α helix when participating to the binding to the viral RNA (Figure 6B,C).

Moreover, the linker between the two zinc fingers is different between HIV-1 and FIV. For HIV 1, it is a seven-residue linker composed mostly of basic residues around a central proline, which plays a role in the relative orientation of the zinc fingers upon binding to the viral RNA (Figure 6). For FIV, this linker is shorter. 

**Figure 7:**
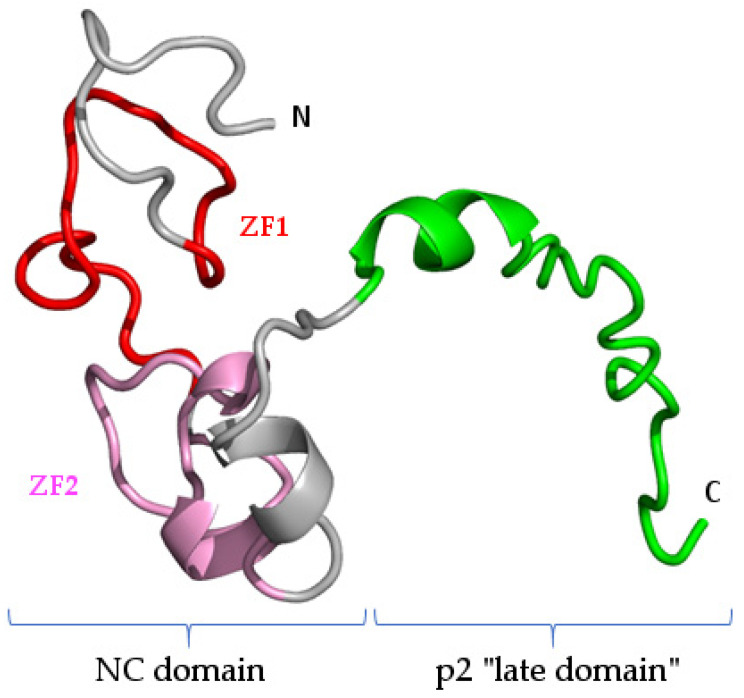
Structure prediction of the FIV p13 subunit, with the NC domain in gray and the “late domain” p2 region in green. Zinc fingers are highlighted in red and pink according to Figure 6. N- and C-terminal extremities are indicated. This structure was extracted from the structure prediction displayed in Figure 5.

No experimental structural data are available for FIV NC, but the de novo prediction of the structure of the Gag polyprotein (Figure 5) suggests the close vicinity of the two zinc fingers of FIV NC even in the absence of RNA (Figure 7, pink and red). Interestingly, this structure prediction also suggests the presence of an α-helix after the second zinc finger in the C-terminal region of FIV NC (Figure 7, gray), which mimics the helix present in the N-terminal region of HIV-1 NC in the presence of the viral RNA (Figure 6C) [68]. 

Thus, it seems that the FIV NC can play a similar role in the interaction with the viral RNA although the spatial distribution of the functional domains as well as the linker between ZF are different from HIV-1 NC. This difference in spatial distribution could be related to the fact that the FIV RNA encapsidation signal (Ψ), characterized by strong secondary structures, shows a different structural arrangement from that of HIV-1 [69,70]. Structural data on the FIV NC domain alone or in complex with its cognate RNA would help to clarify this point.

Mutagenesis studies have demonstrated that mutations in the nucleocapsid of FIV are poorly tolerated and that deletion or modification of either zinc finger leads to significant reduction in virion release or inactivation of the virus [67]. Thus, the zinc fingers of FIV NC are necessary to recruit the viral RNA and represent as such an interesting therapeutic target. Interestingly, recent work demonstrated in vitro the antiviral efficacy of compounds which are ejecting the zinc ions from FIV NC zinc fingers [71], and could represent an interesting therapeutic target. However, it has to be noted that although the structure of the FIV Ψ signals seems conserved across FIV species, their sequence is not conserved [70]. There could therefore be strain-specific determinants of the FIV NC interaction for the Ψ signal, which have not yet been explored but which should be considered for the development of anti-FIV strategies targeting NC.

Concerning the “late domain” function, it has been described for HIV-1 that the “late domain” p6 subunit is able to recruit host proteins from the ESCRT (endosomal sorting complexes required for transport) complexes. These complexes are composed of multiple cellular proteins and allow for the deformation of the cell membrane, the abscission and the release of budding viral particles [72]. The HIV-1 “late domain” p6 subunit contains two conserved motifs, PTAP and YPXnL (where Xn represents one to three variable residues), which bind to two members of the ESCRT family, TSG101 (Tumor Susceptibility Gene 101) and Alix (ALG-2 Interacting Protein X), respectively [66]. These interactions are necessary for the efficient release of HIV-1 viral particles.

It has been shown that the p2 region of FIV p13 contains a PSAP sequence, allowing the interaction with TSG101 and the budding of new viral particles [19,34]. However, the interaction of FIV Gag with Alix in human cells seems to involve a yet unidentified region of Gag, which is independent of the p2 region [66]. This suggests that the “late domain” of FIV Gag is not only composed of the p2 subunit. It should be noted that the “late domain” of HIV-1 has also been suggested to play a role in the selection of the correct viral RNA [8]. Although it has not been demonstrated yet, it is possible that the C-terminal region of FIV p13 possesses such a role in the selection of the correct viral RNA. This is supported by the fact that a FIV Gag polyprotein deleted for this domain demonstrated a transdominant negative phenotype on viral replication [27]. 

**Figure 8:**
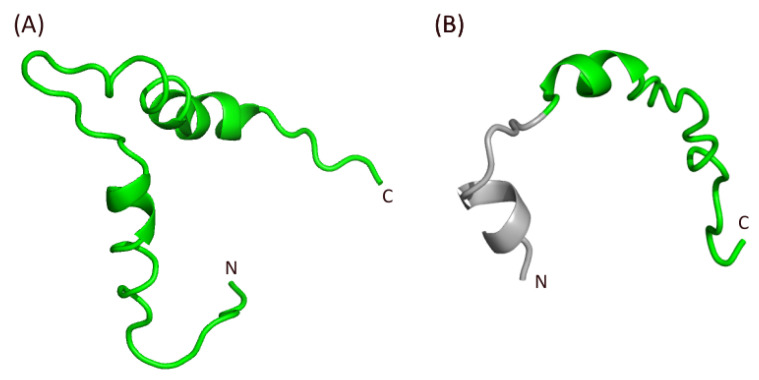
Comparison of the structures of (**A**) the HIV-1 “late domain” p6 subunit (PDB ID 2C55) with (**B**) a close-up view from Figure 7 focusing on the C terminal region of FIV p13, with the C-terminus of the NC domain in gray and the putative “late domain” p2 region in green. N- and C-terminal extremities are indicated for each fragment.

Notably, the structure of the “late domain” of HIV-1 has been shown to contain two α-helices [73] (Figure 8A). Interestingly, the C-terminal region of the p13 subunit of FIV Gag is also predicted to contain two α-helices, but this includes the one in the putative p2 subunit and the one present at the C-terminus of the “NC” region of FIV (Figure 8B, green and gray, respectively). Thus, the medial α-helix of FIV p13 could play a role both for RNA binding (NC function, Figure 7) and RNA selection (“late domain” function, together with the α-helix of the “late domain” distal region of Gag, Figure 8B). This is a further argument to suggest that the FIVp13 subunit needs to combine both the NC domain and the p2 putative fragment to perform “late domain” functions similar to HIV-1 p6, which are therefore not mediated by p2 alone. More experimental evidence will be needed to confirm this point. Thus, acquiring experimental structural data on complete FIV p13 in complex with the viral RNA could be the basis of the development of a specific drug-design strategy.

## 7. Conclusions

Despite the low sequence identity between them, the FIV Gag polyprotein has mostly been envisioned as a functional homologue of HIV-1 Gag, as both led to a similar virion morphogenesis. Structural data that arose from the MA and CA subunits of FIV Gag, which are responsible for the association of the protein to the cell membrane and for the formation of the viral core, respectively, seem to confirm this point. Moreover, the structural prediction of SP is also moving in the same direction. The C-terminal domains of Gag (FIV p13 or HIV-1 NC and p6) are mediating similar functions, including the selection and encapsidation of the species-specific viral RNA into the viral core, and the interaction with cellular partners involved in viral abscission that seem to differ between human and feline cells. However, sequence analysis and structure comparison suggest that the C-terminal extremity of FIV and HIV-1 Gag polyproteins seem to behave differently, with a cooperativity between NC and “late domain” regions for FIV p13 *vs.* independent functional NC and “late domain” subunits for HIV-1. It seems therefore that the evolution of the Gag polyprotein between HIV-1 and FIV is the result of a combination of opposite evolutionary selection processes: a strong selective pressure to conserve the structural and molecular mechanisms involved in virion morphogenesis, associated with a divergence to adapt to species–specific constraints for viral RNA selection, encapsidation, and viral abscission. The conservation of key mechanisms between HIV-1 and FIV, despite millions of years of divergence of viral and host evolution [74], underlines their importance for viral replication and might represent the Achilles’ heel of these lentiviral infections.

## Data Availability

Not applicable.
